# Association between impulsivity and orthorexia nervosa / healthy orthorexia: any mediating effect of depression, anxiety, and stress?

**DOI:** 10.1186/s12888-021-03594-4

**Published:** 2021-12-03

**Authors:** Emmanuelle Awad, Pascale Salameh, Hala Sacre, Diana Malaeb, Souheil Hallit, Sahar Obeid

**Affiliations:** 1 Faculty of Social Sciences, Psychology Department, Lund University, Lund, Sweden; 2 INSPECT-LB: Institut National de Santé Publique, Épidémiologie Clinique et Toxicologie-Liban, Beirut, Lebanon; 3 Faculty of Pharmacy, Lebanese University, Hadat, Lebanon; 4 University of Nicosia Medical School, Nicosia, Cyprus; 5 School of Pharmacy, Lebanese International University, Beirut, Lebanon; 6 Life Sciences and Health Department, Paris-Est University, Paris, France; 7 Faculty of Medicine and Medical Sciences, Holy Spirit University of Kaslik (USEK), Jounieh, Lebanon; 8 Research Department, Psychiatric Hospital of the Cross, Jal Eddib, Lebanon; 9 Psychology Department, Faculty of Arts and Sciences, Holy Spirit University of Kaslik (USEK), Jounieh, Lebanon

**Keywords:** Orthorexia nervosa, Healthy orthorexia, Anxiety, Depression, Stress, Impulsivity

## Abstract

**Background:**

There is a lack of studies investigating the possible mediating role of psychological factors, such as depression, anxiety and stress on orthorexic eating behaviors. Given that personality attributes might affect the manifestation of psychological disorders, it was hypothesized that depression, anxiety and stress mediate the relationship between impulsivity-related traits and orthorexic eating, noting that previous research had evaluated the role of depression as a mediator between impulsivity and other pathological eating behaviors. The study objectives were to explore the mediating effect of depression, anxiety, and stress, on impulsivity and orthorexia nervosa, and healthy orthorexia.

**Methods:**

This cross-sectional study conducted between July and December 2019 recruited 519 Lebanese adults from seven community pharmacies randomly selected from a list provided by the Lebanese Order of Pharmacists.

**Results:**

Our results showed that depression and anxiety were positively correlated with ON. We also found a notable gender difference in findings: higher anxiety and female gender were significantly associated with higher TOS healthy orthorexia, while higher stress was associated with lower TOS healthy orthorexia. Finally, higher urgency was associated with lower TOS healthy orthorexia, while higher perseverance was significantly associated with higher TOS healthy orthorexia. Depression and anxiety partially mediated the association between perseverance and ON while anxiety and stress partially mediated the association between urgency and healthy orthorexia.

**Conclusion:**

Our study suggests that depression, anxiety and stress play a mediating role between impulsivity and orthorexia nervosa/healthy orthorexia. Our findings provide a ground for future investigations of impulsive behaviors, psychopathology, and orthorexia in different populations.

**Supplementary Information:**

The online version contains supplementary material available at 10.1186/s12888-021-03594-4.

## Background

Orthorexia Nervosa (ON) involves a dysfunctional preoccupation with consuming foods perceived as healthy and an extremely strict adherence to self-imposed diet restrictions [1]. Key elements of ON are “(a) obsessive focus on dietary practices believed to promote optimum well-being through healthy orthorexia (with inflexible dietary rules, recurrent and persistent preoccupations related to food, compulsive behaviors); and (b) consequent, clinically significant, impairment (e.g. medical or psychological complications, great distress, and/or impairment in important areas of functioning)” [2]. Unlike those with Anorexia Nervosa, individuals with ON are concerned with regulating food quality rather than their weight. ON can cause significant distress if the dietary rules are broken, as well as physical, psychological, and social impairment [3]. People with ON can become severely underweight, develop anxiety disorders and depression, and socially isolate themselves to avoid exposure to what they consider as impure nutrition [3]. Despite its adverse effects, ON is not included as an eating disorder in the Diagnostic and Statistical Manual of Mental Disorders- 5th edition (DSM-5) as of now [4]. Several diagnostic criteria of ON overlap with symptoms of other psychological disorders [2], such as obsessive-compulsive disorder (OCD) [5, 6] and Anorexia Nervosa (AN) [7], triggering discussions about its categorical classification [8]. Having said that, the essence differs between ON and AN: the main concern for orthorexic eating behaviors are health and purity while the core of AN is weight loss and food quantity [7]. On the other hand, some authors assume two different subtypes of orthorexia: ON and healthy orthorexia, characterized by the non-pathological fixation on consuming healthy foods. Key elements of healthy orthorexia are a “healthy interest in diet, healthy behavior with regard to diet, and eating healthily as part of one’s identity” [9]. However, little research exists on its traits and associated factors, with the majority of the literature focusing on ON.

### Orthorexia measures of assessment

Various psychometric tools to measure ON were used, such as the Bratman Orthorexia test (BOT), Düsseldorfe Orthorexia scale (DOS), and different validated versions of the ORTO-15 and ORTO-11, the most used questionnaires to assess ON. The ORTO-15 received heavy criticism for its significant psychometric limitations [10]; thus, researchers developed alternative measures, such as the Teruel Orthorexia Scale (TOS), which includes items measuring both ON and Healthy Orthorexia (HeOr) [11]. ON consists of extreme focus on food intake and preoccupation with designing and following the optimal diet. Adherence to the diet is done by following strict dietary rules, subsequently causing distress and impairment (Cena et al., 2019). On the other hand, HeOr is the non-pathological dimension of orthorexia, which consist of placing importance on a healthy diet and lifestyle. Therefore, the prevalence varies depending on the instrument used and the population at study. In 2004, one of the first studies in Italy used ORTO-15 and showed a prevalence of 6.9% in a population of 404 students [12]. Another one using BOT in a sample of 283 dieticians found that 34.9% of the population had a high risk of ON [13]. A German study using the DOS on 446 university students reported a prevalence of 3.3%, with a 9.0% risk of developing ON [14]. A recent study showed an orthorexia prevalence of 2.6% in Polish adults [15].

A previous study showed that the onset of restrictive eating disorders occurred earlier and these patterns were less severe for males as opposed to females [16, 17]. Females also exhibit more maladaptive eating symptomatology than men [18]. On the other hand, findings show that sex-specific hormones make women more vulnerable than men to suffer from maladaptive eating patterns at certain ages [19]. An association between education level and maladaptive eating patterns was found, with women who have lower education being more likely to suffer from eating disorders [20]. Differences were also found among men and women in their BMI: women with restrictive eating patterns had lower BMI compared to women who don’t restrict, while men with restrictive eating patterns had a higher BMI compared to men who don’t restrict [21]. These findings suggest the presence of gender differences in the presentation of maladaptive eating patterns.

### Impulsivity and eating styles

Impulsivity has been used as a factor to assess and predict neuropsychological disorders [22]. The neurological processes behind impulsivity are clinically relevant to multiple psychological disorders, including eating disorders and OCD [23]. Research also found that latent neurobiological functions of impulsivity involve a broad range of psychological disorders, including OCD, eating disorders, depression, and anxiety disorders [24]. Furthermore, predisposing factors for depression and anxiety included impulsivity [25], while stress predicted risky and impulsive behaviors [26]. The neurological links between impulsivity, psychological disorders (including depression, anxiety, and stress), and obsessive-compulsive and eating disorders, suggest a possible relationship involving these variables.

Impulsivity encompasses different traits, each referring to a behavior: urgency (careless behavior), premeditation or purpose (the extent of planning), perseverance (determination), and risk (level of sensation seeking) [27]. Levels of impulsivity can differ among individuals with eating disorders and affect the way it is manifested [28]. The number of studies evaluating impulsivity, ON and healthy orthorexia is limited. Patients with eating disorders showed lower perseverance and higher urgency [29]. High harm avoidance (low risk-seeking) is associated with all eating disorders [30, 31]. In addition, a previous study had revealed that impulsivity described a process in which high levels of negative urgency predicted more disordered eating, which would help manage negative affect (depression) [32]. As for healthy orthorexia, a recent study found that impulsivity was negatively associated with diet quality (fruit and vegetables, meat and poultry, processed meat, dairy products, milk-based desserts, and starchy foods) [33].

### Mental health and orthorexia

Around half of the individuals who have restrictive eating patterns meet the criteria for Generalized Anxiety Disorder (GAD), and 15% of those who have eating disorders also have social phobia [34]. Major Depressive Disorder (MDD) and anxiety disorders, including GAD, were found as possible risk factors for developing ON [35]. As for healthy orthorexia, studies have long linked healthy diets, particularly those rich in fruits, vegetables, fish and whole grains, with a reduced risk of depression [36]. Furthermore, a previous study showed that ON was only partially associated with psychological problems such as depression, anxiety and stress, and this effect was moderated by healthy orthorexia in a German sample [37].

### Orthorexia in Lebanon

Lebanon is a Middle-Eastern country, which has gone through political and war events that have affected the mental wellbeing of its residents, with high rates of depression [38], anxiety [39] and alexithymia [40] shown in recent studies. Different situations occurred in Lebanon (Lebanese civil war, protestation since 17 October 2019, Beirut Blast on 4 August 2020, the economic crisis in parallel with the COVID-19 pandemic) had many negative consequences on the mental health of the Lebanese population [41]. More particularly, in a sample of Lebanese adults, a total of 75.2% had a risk for developing ON [42]; yet, it is not commonly known or considered a disorder in this population. This number might be unrealistic since the validated ORTO-15 scale had low psychometric properties, which yielded a high prevalence of ON [43]. A recent study revealed an orthorexia prevalence of 8.4% in Lebanese adults using the Dusseldorf Orthorexia Scale [15]. Another study revealed that individuals from a Lebanese sample who met the criteria for ON experienced less psychological distress [44], but the literature connecting psychological distress to ON is scarce. A recent study among Lebanese medicine students found that higher anxiety was associated with lower ON tendencies and behaviors [44]. It was also found that behavioral problems related to eating habits were associated with impulsivity in the Lebanese population [45].

### Objective of the study and rationale

**Fig. 1:**
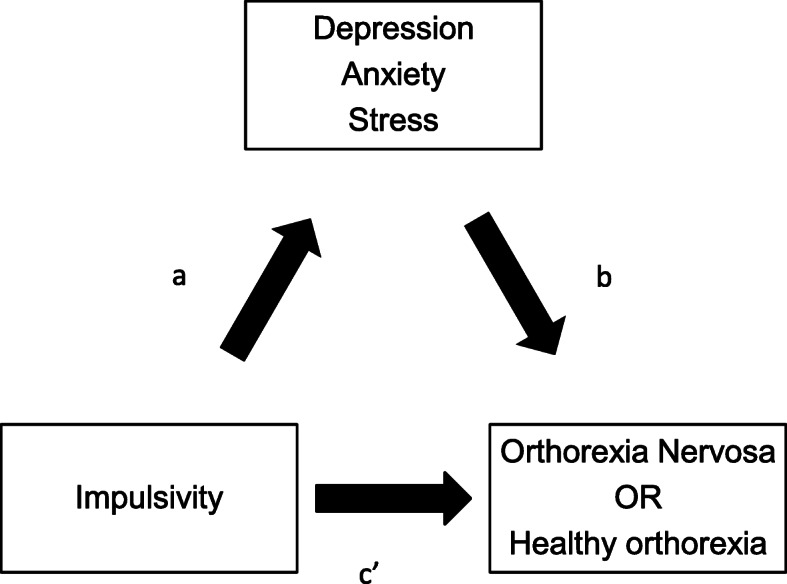
**a** Supposed relation between impulsivity and depression, anxiety and stress; **b** Supposed relation between depression, anxiety, stress and orthorexia nervosa/heathy orthorexia; **c’** Relation between impulsivity and orthorexia nervosa/heathy orthorexia

Overall, the literature presents correlations with no inference of causation of either directions, or exploration of possible mediations for orthorexia. Given that previous research found that impulsivity, depression and anxiety symptoms may overlap and be associated with certain brain structures [46], it was hypothesized that depression, anxiety, or stress mediate the relationship between impulsivity-related traits and orthorexia dimensions in order to further explore the nature of these relationships (Fig. 1); noting that previous research had evaluated the role of depression as a mediator between impulsivity and eating disorders, however it did not include orthorexia [42].

Therefore, the objective of this study was to evaluate the association between impulsivity and orthorexia nervosa/healthy orthorexia after adjusting over sociodemographic variables and explore the mediating effect of depression, anxiety, and stress, on impulsivity and ON/healthy orthorexia, using the Teruel Orthorexia Scale (TOS) was used to measure orthorexia nervosa and healthy orthorexia [34]. Particularly, the following hypotheses were concentrated on:

1. Correlations exist between impulsivity traits and orthorexia, sociodemographic variables and orthorexia, and mental health variables (depression, anxiety and stress) and orthorexia.
2. Different correlations exist between impulsivity traits, sociodemographic variables and mental health variables (depression, anxiety and stress) and both dimensions of orthorexia ON and HeOr.
3. Mental health variables (depression, anxiety and stress) mediate the relationship between impulsivity traits (urgency, premeditation, perseverance and sensation seeking) and ON or HeOr.

## Methods

The sample comprised 519 adults (*n* = 283, 56.0% women) with a median age of 33.50 years (mean: 36.02, standard deviation: 14.20, range: 18–75) and a median BMI of 24.21 kg/m^2^ (mean: 24.41, standard deviation: 4.32, range: 17.01–37.78). More than half of the sample had university education level (*n* = 252, 53.1%) and reported to be unmarried (*n* = 264, 51.6%) (Table 1).

**Table 1:** Sociodemographic characteristics of the sample (*N* = 519)

Variable	N (%)
**Gender**	
Male	222 (44.0%)
Female	283 (56.0%)
**Marital status**	
Single	232 (45.4%)
Married	247 (48.3%)
Widowed	17 (3.3%)
Divorced	15 (2.9%)
**Education level**	
Primary	30 (6.3%)
Complementary	74 (15.6%)
Secondary	119 (25.1%)
University	252 (53.1%)
**Monthly income**	
No income	138 (29.5%)
< 1000 USD	148 (31.6%)
1000–2000 USD	133 (28.4%)
> 2000 USD	49 (10.5%)

### Procedure

This cross-sectional study conducted between July and December 2019 recruited a sample of Lebanese adults from seven community pharmacies randomly selected from a list provided by the Lebanese Order of Pharmacists. Every person 18 years and above entering a pharmacy was eligible and encouraged to participate in the study. Well-trained personnel explained the study objectives to participants. Of the 700 questionnaires distributed, 520 (74.28%) were completed and collected back. All methods were carried out in accordance with relevant guidelines and regulations.

### Sample size calculation

The G-power software calculated a minimum sample of 395 participants, based on an effect size f2 = 2%, an alpha error of 5%, a power of 80%, and taking into consideration 20 factors to be entered in the multivariable analysis.

### Questionnaire and variables

The self-administered questionnaire was anonymous and available in Arabic, the mother tongue in Lebanon. It required 15–20 min to complete approximately.

The first part clarified socio-demographic characteristics: age, gender, level of education, and monthly income, in addition to weight (in kg) and height (in meters) to calculate the Body Mass Index (BMI).

The second part included the ***Teruel Orthorexia Scale (TOS)*** [11], a relatively new tool consisting of 17 items rated on a 4-point Likert scale ranging from 0 (completely disagree) to 3 (completely agree). Validated in Lebanon [47, 48], this scale yields two subscale scores with related statements: HeOr (e. g., “My interest in healthy food is an important part of the way I am, of how I understand the world”) and ON (e.g., “I am concerned about the possibility of eating unhealthy foods”), with higher scores indicating higher orthorexia nervosa/healthy orthorexia.. Reliability was very good for both subscales (α_Cronbach_ = 0.87). Permission was obtained from Dr. Juan-Ramon Barrada and Dr. Maria Roncero to use the scale in this study. The self-administered ***I-8 scale*** assessed urgency (e. g., “To make myself feel better, I sometimes do things I regret later.”), premeditation (e. g., “I usually think carefully before I do anything”), perseverance (e. g., “I allocate my time well so that I can complete tasks on time.”), and sensation risk (e. g., “I’m ready to take risks.”). All items are rated from 0 (doesn’t apply at all) to 5 (applies completely), with higher scores indicating higher impulsivity. In this study, the Cronbach’s alpha values of the subscales were as follows: Perseverance: 0.802; urgency: 0.796; premeditation: 0.815; and sensation seeking: 0.613 [49].

Depression was assessed using the ***Hamilton Rating Scale for Depression (HAM-D)***, validated in Lebanon [50]. This scale includes 17 items such as depressed mood, feelings of guilt, suicide, insomnia, work and activities, somatic symptoms, weight and insight. Each item refers to specific depression symptomatology; it is rated from 0 to 2–4, depending on the item, 2–4 being the most severe (α_Cronbach_ = 0.871). Scores < 10 indicate no depression, 10–13 mild depression; 14–17 mild to moderate depression; > 17 moderate to severe depression. The questionnaire also consisted of the ***Hamilton Anxiety Rating Scale*** that assessed the level of anxiety. This tool validated in the Lebanese population [51] includes 14 items describing conditions such as anxious mood, insomnia, somatic symptoms, tension and respiratory symptoms. The items are rated from 0 to 4, 4 being the most severe (α_Cronbach_ = 0.917). Stress was measured using the ***Beirut Distress Scale (BDS10)****.* This 10-item tool has been recently validated in Lebanon to assess psychological distress [52]. The scale included statements such as: “I lost the desire to learn”, “My mood changes for tiny matters” and “I worry about little things”. Items are rated over a four-point Likert scale, with higher scores reflecting higher psychological distress (α_Cronbach_ = 0.899).

### Forward and back translation procedure

The translation procedure was done according to previous methods described in the literature [53–55]. A clinical psychologist, whose native language is Arabic and fluent in English, performed the forward translation for the I-8 scale. Two psychiatrists, a medical professional writer and the primary investigators verified the Arabic translated version. A backward translation was then performed by another psychologist, fluent in Arabic, and unfamiliar with the concepts of the scales. The back-translated English questionnaire was subsequently compared to the original English one by the principal investigator to discern discrepancies and solve any inconsistencies between the two versions. The process of forward and back-translation was repeated until all ambiguities disappeared. The questionnaire was pilot-tested on a sample of 20 participants before the data collection was officially started. The results of the pilot sample test were not included in the final datasheet.

### Statistical analysis

A factor analysis was initiated to confirm the legitimacy of the construct of the I-8 scale in our sample using the FACTOR program; the Pearson correlation matrix was used, with a parallel analysis procedure for determining the number of factors to be retained. A varimax rotation was used since the items of the I-8 scale were not highly correlated. The Kaiser-Meyer-Olkin (KMO) measurement of sampling adequacy and Bartlett’s sphericity test were appropriate. The factors retained corresponded to Eigenvalues greater than one. Statistical analysis was performed using SPSS software, version 23. Data were screened for missing and unrealistic values (e.g., aged 5, or BMI above 60 kg/m2 or less than 16 kg/m2) that were restored when possible, or otherwise considered missing. The normality of distribution of the TOS OrNe and OrHe scores were confirmed via a calculation of the skewness and kurtosis; values for asymmetry and kurtosis between − 2 and + 2 are considered acceptable in order to prove normal univariate distribution [56]. These conditions consolidate the assumptions of normality in samples larger than 300 [57]. Accordingly, the Student t-test was used to check for an association between TOS ON and healthy orthorexia scores and dichotomous variables (i.e., gender and marital status) while the ANOVA test was used to compare the TOS ON and healthy orthorexia means between three or more means (i.e., education level and monthly income). The Pearson correlation test was used to correlate two continuous variables (i.e., age, BMI, depression, anxiety, stress, and impulsivity). A multivariate analysis of covariance (MANCOVA) was carried out to compare multiple measures (TOS ON and healthy orthorexia scores taken as dependent variables) between the different factors, taking into account potential confounding variables: age, gender, education level, monthly income, and BMI.

### Mediation analysis

The PROCESS SPSS Macro version 3.4 model four [58] was used to calculate three pathways. Pathway A determined the regression coefficient for the effect of impulsivity on depression/anxiety/stress, Pathway B examined the association between depression/anxiety/stress and ON/healthy orthorexia, independent of impulsivity, and Pathway C′ estimated the total and direct effect of impulsivity on ON/healthy orthorexia. Pathway AB calculated the indirect intervention effects (Fig. 1). To test the significance of the indirect effect, the macro generated bias-corrected bootstrapped 95% confidence intervals (CI) [58]. A significant mediation was determined if the CI around the indirect effect did not include zero [58]. The covariates that were included in the mediation model were those that showed significant associations with ON in the bivariate analysis. *P* < 0.05 was considered significant.

## Results

### Description of the scores

The mean scores for the TOS, depression, anxiety, and stress scores were as follows: TOS ON 7.75 ± 5.62, TOS healthy orthorexia 10.57 ± 6.04, depression 6.09 ± 7.27, anxiety 12.86 ± 10.52, and stress 17.34 ± 4.60.

### Factor analysis

The total sample (*n* = 519) was used for the factor analysis of the I-8 scale items. The KMO value of 0.704 and the significant Bartlett’s sphericity test (*p* < 0.001) ensured the adequacy of the model. All items of the I-8 scale were extracted; the observation of the scree plot and Eigen values higher than 1 suggested a four-factor solution, explaining 81.97% of the total variance. These four factors were labeled perseverance (Factor 1), urgency (Factor 2), premeditation (Factor 3) and sensation seeking (Factor 4) respectively. Finally, the α_Cronbach_ value was acceptable (0.641) (Table 2).

**Table 2:** Factor analysis of the I-8 impulsivity scale using the varimax rotation

Variable	Factor 1	Factor 2	Factor 3	Factor 4
I8–6	0.919			
I8–5	0.897			
I8–2		0.926		
I8–1		0.904		
I8–3			0.994	
I8–4			0.791	
I8–7				0.862
I8–8				0.844
**Percentage of variance explained**	38.51	21.25	13.56	8.65
**Cronbach’s alpha**	0.802	0.796	0.815	0.613

Factor 1 = perseverance; Factor 2 = urgency; Factor 3 = premeditation; Factor 4 = sensation seeking

### Bivariate analysis

A significantly higher mean TOS healthy orthorexia score was found in females compared to males and in married compared to single participants; whereas, none of these variables was significantly associated with TOS ON (Table 3).

**Table 3:** Bivariate analysis of categorical and dichotomous variables associated with orthorexia nervosa

Variable	TOS orthorexia nervosa	TOS healthy orthorexia
**Gender**		
Male	7.35 ± 5.65	9.70 ± 5.88
Female	8.00 ± 5.66	11.19 ± 6.13
p	0.204	**0.006**
**Marital status**		
Single/divorced/widowed	7.80 ± 5.69	9.80 ± 6.08
Married	7.70 ± 5.55	11.40 ± 5.89
p	0.840	**0.003**
**Education level**		
Illiterate/primary	6.40 ± 5.16	9.43 ± 5.65
Complementary	8.59 ± 6.42	10.20 ± 7.15
Secondary	7.44 ± 5.12	9.58 ± 5.49
University	7.53 ± 5.89	11.24 ± 6.20
p	0.303	0.065
**Monthly income**		
No income	8.11 ± 6.06	10.26 ± 6.47
< 1000 USD	7.34 ± 5.31	10.43 ± 6.15
1000–2000 USD	7.68 ± 5.27	10.94 ± 5.77
> 2000 USD	8.96 ± 6.50	11.86 ± 5.52
p	0.328	0.398

This table presents the mean ± standard deviations of the associations between each independent variable and the orthorexia nervosa/healthy orthorexia scores. The Student t-test was used to compare between orthorexia nervosa/healthy orthorexia scores and gender and marital status, whereas the ANOVA test was used to compare between orthorexia nervosa/healthy orthorexia scores and education level and monthly income

Older age, BMI, anxiety, stress, urgency, and perseverance were significantly but weakly associated with higher TOS ON. Additionally, higher anxiety was significantly, but weakly, associated with higher TOS healthy orthorexia; whereas, higher BMI and perseverance were significantly, but weakly, associated with lower TOS healthy orthorexia (Table 4). It is noteworthy that premeditation and sensation-seeking were not significantly associated with ON and healthy orthorexia.

**Table 4:** Bivariate analysis of continuous variables associated with orthorexia nervosa and healthy orthorexia

Variable	TOS orthorexia nervosa	TOS healthy orthorexia	Age	BMI	Depression	Anxiety	Stress	I8 urgency	I8 premeditation	I8 perseverance	I8 sensation seeking
TOS OrNe	1										
TOS HeOr	.71^a^	1									
Age	.12^a^	.01	1								
Body mass index	.09^c^	-.08^c^	.40^a^	1							
Depression	.03	.02	.07	.13^b^	1						
Anxiety	.29^a^	.15^b^	−.02	.06	.48^a^	1					
Stress	.11^b^	−.04	.12^b^	−.07	.19^a^	.29^a^	1				
I8 urgency	.20^b^	−.07	-.19^a^	−.08	.06	.17^a^	.22^a^	1			
I8 premeditation	.07	−.05	-.16^a^	−.06	−.07	.08	.21^a^	.13^c^	1		
I8 perseverance	.14^a^	-.10^b^	-.16^a^	.01	−.02	.10^c^	.16^a^	.18^a^	.57^a^	1	
I8 sensation seeking	−.06	.01	−.02	.02	.01	-.11^c^	-.10^c^	.05	-.26^a^	-.32^a^	1

^a^*p* < 0.001; ^b^ *p* < 0.01; ^c^ *p* < 0.05; the Pearson correlation test was used to correlate between orthorexia nervosa/healthy orthorexia scores and continuous variables; numbers included in this table represent the correlation coefficients (r)

### Multivariate analysis

Higher depression and anxiety were significantly associated with higher ON, with none of the impulsivity subscales being associated with ON. Moreover, higher anxiety and female gender were significantly associated with higher TOS healthy orthorexia, while higher stress was significantly associated with lower TOS healthy orthorexia. Finally, higher urgency was associated with lower TOS healthy orthorexia, while higher perseverance was significantly associated with higher TOS healthy orthorexia (Table 5).

**Table 5:** Multivariate analysis (MANCOVA) taking TOS orthorexia nervosa and healthy orthorexia as dependent variable and each impulsivity subscale as an independent variable

	TOS orthorexia nervosa				TOS healthy orthorexia			
**Model 1: I8 urgency score taken as an independent variable.**								
**Variable**	**Beta**	**p**	**95% CI**	**Partial Eta Squared**	**Beta**	**p**	**95% CI**	**Partial Eta Squared**
Depression	−0.124	**0.005**	−0.211- -0.038	0.019				
Anxiety	0.187	**< 0.001**	0.128–0.246	0.087	0.145	**< 0.001**	0.081–0.210	0.046
Stress					−0.162	**0.018**	−0.295- -0.028	0.014
I8 urgency	0.120	0.599	−0.328-0.568	0.001	−0.668	**0.007**	−1.156- -0.180	0.017
Gender (females vs males*)					1.416	**0.018**	0.245–2.587	0.014
**Model 2: I8 premeditation score taken as an independent variable.**								
**Variable**	**Beta**	**p**	**95% CI**	**Partial Eta Squared**	**Beta**	**p**	**95% CI**	**Partial Eta Squared**
Depression	−0.123	**0.006**	−0.211- -0.036	0.019				
Anxiety	0.191	**< 0.001**	0.131–0.251	0.089	0.142	**< 0.001**	0.076–0.207	0.043
Stress					−0.178	**0.01**	−0.315- -0.042	0.016
I8 premeditation	−0.238	0.358	−0.747-0.271	0.002	0.426	0.133	−0.130-0.983	0.006
Gender (females vs males*)					1.348	**0.025**	0.170–2.525	0.012
**Model 3: I8 perseverance score taken as an independent variable.**								
**Variable**	**Beta**	**p**	**95% CI**	**Partial Eta Squared**	**Beta**	**p**	**95% CI**	**Partial Eta Squared**
Depression	−0.126	**0.004**	−0.213- -0.040	0.02				
Anxiety	0.186	**< 0.001**	0.126–0.246	0.084	0.140	**< 0.001**	0.075–0.206	0.042
Stress					−0.166	**0.016**	−0.301- -0.032	0.014
I8 perseverance	−0.456	0.061	−0.933-0.021	0.009	0.603	**0.023**	0.082–1.125	0.013
Marital status (married vs single*)					1.472	**0.029**	0.155–2.789	0.012
**Model 4: I8 sensation seeking score taken as an independent variable.**								
**Variable**	**Beta**	**p**	**95% CI**	**Partial Eta Squared**	**Beta**	**p**	**95% CI**	**Partial Eta Squared**
Depression	−0.131	**0.003**	−0.218- -0.044	0.021				
Anxiety	0.191	**< 0.001**	0.131–0.251	0.088	0.132	**< 0.001**	0.067–0.198	0.037
Stress					−0.183	**0.007**	−0.317- -0.050	0.018
I8 sensation seeking	0.039	0.880	−0.465-0.542	0.001	0.085	0.761	−0.464-0.634	0.001
Gender (females vs males*)					1.353	**0.024**	0.180–2.525	0.012

### Mediation analysis of orthorexia nervosa

**Fig. 2:**
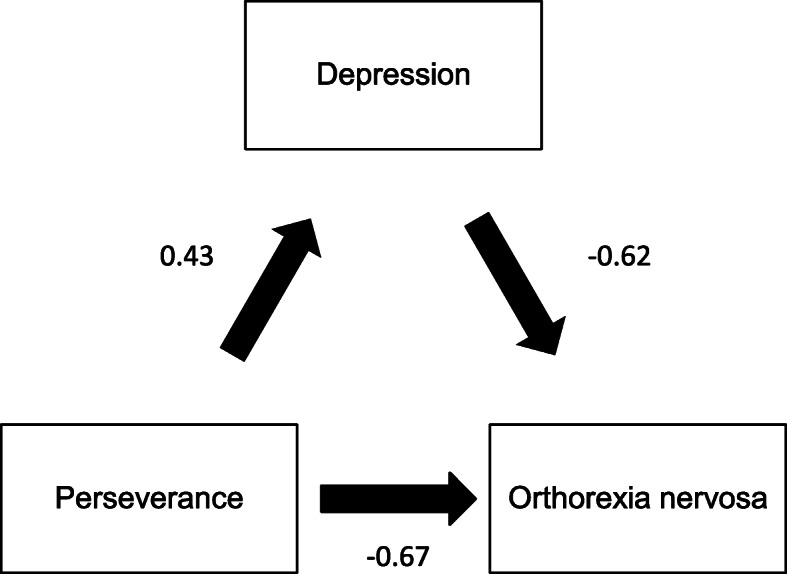
Relation between perseverance and orthorexia nervosa, mediated by depression (numbers in the figure refer to standard coefficients)

**Fig. 3:**
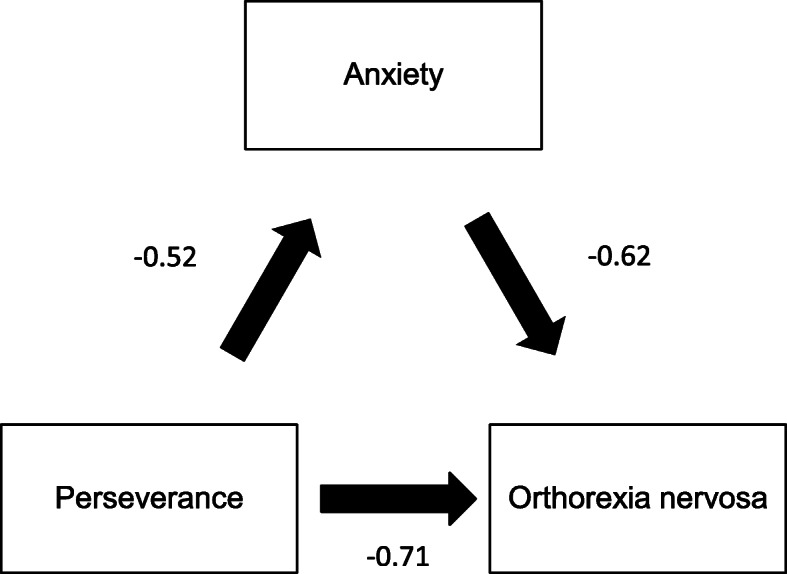
Relation between perseverance and orthorexia nervosa, mediated by anxiety (numbers in the figure refer to standard coefficients)

Depression and anxiety partially mediated the association between perseverance and ON by 8.08% (Fig. 2) and 14.65% (Fig. 3), respectively*.* Stress did not mediate the association between impulsivity and ON.

### Mediation analysis of healthy orthorexia

**Fig. 4:**
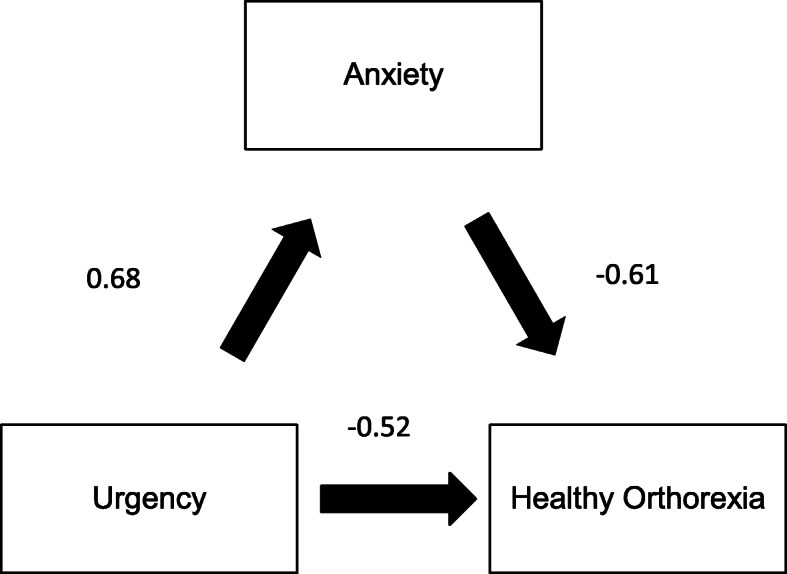
Relation between urgency and healthy orthorexia, mediated by anxiety

**Fig. 5:**
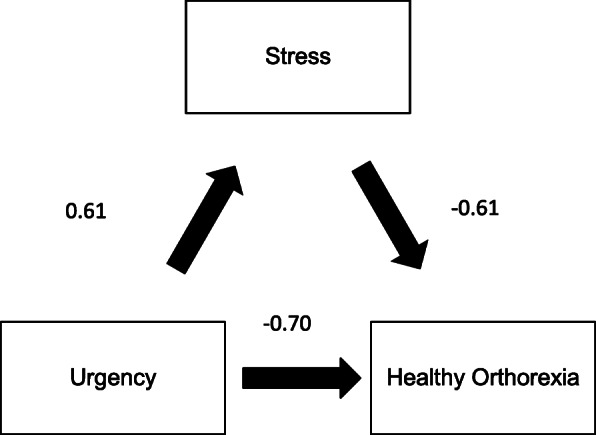
Relation between urgency and healthy orthorexia, mediated by anxiety

Depression did not mediate the association between impulsivity and healthy orthorexia. Anxiety and stress partially mediated the association between urgency and healthy orthorexia by 14.96% (Fig. 4) and 15.12% (Fig. 5), respectively. All details regarding the mediation analysis can be found in Supplementary Tables 1 and 2.

## Discussion

Our study showed that higher depression and higher anxiety were significantly associated with higher orthorexia nervosa, whereas the female gender and higher anxiety were significantly associated with higher TOS healthy orthorexia. Present findings also showed that higher stress was significantly associated with lower TOS healthy orthorexia. In addition, depression and anxiety played a partial mediating role between perseverance and ON, while anxiety and stress partially mediated the relationship between urgency and healthy orthorexia.

### Sociodemographic characteristics and orthorexia

Our results showed that the female gender was significantly associated with healthy orthorexia, in agreement with those of previous findings revealing that women scored higher on healthy orthorexia [59]. A previous study had found a correlation between the female gender and restrained eating, but its relation with healthy orthorexia was insignificant [59], while another did not find any significant differences between genders regarding healthy orthorexia [9]. Women reported more orthorexic behaviors than men cross-culturally [60], as they are under considerably higher societal pressure to follow a restrictive diet and health-based behaviors than males [61]. The reason behind a higher prevalence of restrictive eating disorders among women is clear: the ideal thin body type [62]. However, this may not apply in the case of orthorexic eating behaviors, as the objective is not weight loss but maintaining a strictly healthy diet. A previous study showed that women are more likely to maintain health through good dietary habits while men do so through physical exercise [63]; this could explain why women in our sample present more orthorexic behaviors.

### Depression, anxiety, stress, and orthorexia

Higher depression was associated with higher ON in the present study. These findings are consistent with previous studies that showed a higher prevalence of ON among individuals with depressive traits [64]. It can be hypothesized that orthorexic behavior can result in obsessive eating habits and lead to social isolation, which is in line with behavior associated with depression: depressive symptoms could be manifested by decreased interest in almost all activities [65]. If this line of thought is followed through, the pattern observed by clinicians that orthorexia is indeed accompanied by psychological distress and strain can be supported [64].

Furthermore, higher anxiety scores in our study were associated with both ON and healthy orthorexia, consistent with previous findings showing that anxiety predicted orthorexic behaviors [66]. Also, psychological fear and anxiety were associated with restrictive eating patterns [67], explained by the high levels of physical and health-related anxiety in individuals with ON [68] [69]. ,This outcome is coherent, as orthorexia consists of a preoccupation with eating healthy foods, although the process is extreme and causes anxiety in ON. On the other hand, restrictive eating patterns such as orthorexia can be motivated by anxiety related to health and possible detrimental outcomes from a poor diet or the consumption of unfavorable foods [70]. It is important to mention that anxiety occurs among individuals with maladaptive eating patterns such as ON as well as individuals with normal eating patterns such as healthy orthorexia but levels of anxiety differ [7], which supports our present findings.

Our results also showed that higher levels of stress were associated with lower TOS healthy orthorexia, which is similar to previous studies showing that higher stress was associated with pathological and restrictive eating behaviors [71] and negatively correlated with healthy orthorexia, an adaptive eating lifestyle. Another study confirmed that people with higher scores of ON eating behaviors, suggesting pathological occupation with maintaining a healthy diet, have higher levels of anxiety, stress, and depressive symptoms [70], which could a negative relationship between stress and healthy orthorexia behaviors. It is hypothesized that the preoccupation and worries about eating unhealthy foods and of the effect of food quality and composition on physical or emotional health or both, can lead to higher levels of stress; it is also implied by items of the TOS such as “I am concerned about the possibility of eating unhealthy foods” (Barrada & Roncero, 2019). Furthermore, concern with the quality of one’s diet is present in both orthorxia nervosa and healthy orthorexia, which can be assumed to cause stress or be the result of a certain stress level; however, we can hypothesize that a certain stress level can cause adaptive behaviors such as healthy orthorexia. Stress can be placed on a continuum of different levels, which trigger behaviors that can be adaptive or maladaptive [71]. This can be examined in future research about differences in stress levels and eating patterns.

### Impulsivity and healthy orthorexia

Higher urgency, associated with careless behavior, was correlated with lower TOS healthy orthorexia in our study. Healthy orthorexia is the non-pathological eating patterns [7], while urgency is linked to eating disorders [62]. Additionally, higher perseverance was associated with higher TOS healthy orthorexia in our study. Patients with eating disorders reported lower perseverance scores than those with restrictive behaviors [63]. Healthy orthorexia is a well-adjusted way of eating; results from eating disorders can be used as a reference for the anticipated associated variables with healthy orthorexia: we might hypothesize that impulsivity trait levels might differ between pathological and adaptive eating patterns. Healthy orthorexia is characterized by the adaptive determination to consume healthy foods, which could be comparable to perseverance trait, as opposed to pathological eating, which is predicted by the inability to inhibit emotional responses associated with pathological eating behaviors [69].

### Mediation between impulsivity and ON

Our results showed that depression and anxiety were partial mediators between perseverance and ON. A previous study had confirmed that anxiety mediated between ON and cultural expectations, but not personality traits, such as elements of impulsivity [69]. Perseverance, the persistently pursuing a goal despite adverse outcomes, is associated with less ON scores, which is inconsistent with the proposed diagnostic criteria of ON, where individuals continuously follow dietary rules despite physical, psychological, and social repercussions [3]. Another study showed that restriction-centered eating disorders are associated with low levels of perseverance and confirmed the predictive role of impulsivity traits in eating disorder [16]. Whether impulsivity-related traits affect the outcome, namely ON, might depend on the levels of depression and anxiety of each individual.

### Mediation between impulsivity and healthy orthorexia

In the present study, anxiety and stress were both partial mediators between urgency and healthy orthorexia. This impulsivity trait, i.e., urgency, is inconsistent with healthy orthorexia that focuses on maintaining a healthy diet. The partial mediating role played by psychological factors, such as anxiety and stress, might suggest that additional determinants could affect the pathway between having an eating disorder (ON) or a normal pattern (healthy orthorexia) that could account for a more significant effect between impulsivity and ON. Psychological variables could influence whether levels of impulsivity-related traits contribute to some maladaptive or adaptive eating patterns. Our findings could determine partial mediation, which suggests the intervention of additional elements not discussed within our model. Furthermore, we were not able to find significant results with impulsivity, perseverance, purpose, and risk as independent variables, and depression, anxiety, and stress as mediators with ON and healthy orthorexia, highlighting the need for further research about ON, healthy orthorexia, and their associated variables.

### What is already known on this subject?

Healthy orthorexia is generally considered equivalent to ON in the literature, despite evidence proving orthorexia is bidimensional and encompasses both healthy and pathological aspects. Similarly to ON, no diagnostic criteria or clear set of signs other that previously suggestions exist for healthy orthorexia, however, it is defined as non-disordered commitment to consuming healthy foods. Currently, ON is not qualified as an eating disorder due to insufficient clinical pertinence, therefore, little is known about healthy orthorexia other than information based on knowledge of ON.

### What does this study add?

Our study provides insight on impulsivity, orthorexia dimensions and psychological disorders, as well as socio-demographic correlates and mediating relationships. Considering that orthorexia is a newly emerging eating disorder that has not yet been included as a legitimate pathology, along with the fact that healthy orthorexia has not been separated from ON in many studies, our findings provide a pathway for further investigations regarding impulsive behaviors, psychopathology, and orthorexia (healthy orthorexia and ON) in different populations.

### Clinical implications

Our study adds to the limited body of research revolving around the relationship between impulsivity and ON, impulsivity and healthy orthorexia, the presence of a mediating role played by depression, anxiety, and stress, and ON and healthy orthorexia in general. Our results emphasize the importance of impulsivity-related traits in ON; further research might evaluate ON as a consequence of impulsivity in clinical settings. Understanding this correlation and mediation in eating disorders would help develop treatment and preventive intervention guidelines. Moreover, understanding the personality traits of people with ON allows improving the prediction of people at risk of developing ON. Identifying problematic features of this disorder can also be translated into improved interventions. Our findings also highlight the possible comorbidities of ON. Pathological eating patterns are associated with depression, anxiety, and stress, which legitimatizes its relation with ON and might give clinicians motivation to observe it more carefully among patients.

### Limitations and strengths

Our study has some limitations. Causation cannot be inferred due to its cross-sectional design. Second, the TOS and I-8 scales have yet to be validated to the Lebanese population; however, both were translated to Arabic for this study. Information bias might exist, as participants may have misinterpreted a question, thus decreasing the validity of the response. The scarcity of studies exploring the mediating role of psychological factors between impulsivity and orthorexia limited our discussion; hence, it was based on hypotheses. Although significant, the correlations obtained were weak. Residual confounding bias is also possible since not all factors associated with orthorexia dimensions were taken into consideration in this study. Religion was not taken into consideration in this study; the majority of the Lebanese population is composed of Muslims who may follow different dietary habits than Christians. Participants have not been screened for eating disorders before the start of the data collection. The influences of any cultural and linguistic differences between Spanish and Lebanese populations were not taken into consideration, which might have changed the results of this study. If the Bonferroni correction were to be applied to the statistical analysis, some variables that have been considered to be significantly associated with the dependent variables (such as BMI and stress in case of orthorexia nervosa and gender, BMI and perseverance in case of healthy orthorexia) must have been removed. Thus, these variables are unstable, warranting further confirmation in future studies. No evaluation of the overlap in the definition and content of the construct measured in the TOS was done; future studies should tackle this idea to compare the Spanish and Lebanese populations in this regard. On the other hand, the random sampling method used to select our participants allows the generalization of our results to the Lebanese population.

## Conclusion

The purpose of our study was to investigate possible correlations and mediation roles between impulsivity traits, mental health traits and both Orthorexia dimensions. Significant differences were found between ON and HeOr, and gender and relationship status, as well as significant correlations between impulsivity traits, mental health traits and ON or HeOr. Also, our study showed that depression and anxiety played a partial mediating role between perseverance and orthorexia nervosa, while anxiety and stress partially mediated the relationship between urgency and healthy orthorexia. Our findings provide a ground for future investigations of impulsive behaviors, psychopathology, and orthorexia in different populations.

## Supplementary Information

**Additional file 1.**

## Data Availability

Data cannot be shared publicly because of the restrictions of the ethics committee. Data are available upon a reasonable request to the corresponding author for researchers who meet the criteria for access to confidential data.

## Abbreviations

HeOr: Healthy orthorexia
TOS: Teruel Orthorexia Scale
ON: Orthorexia Nervosa
DSM-5: Diagnostic and Statistical Manual of Mental Disorders- 5th edition
OCD: Obsessive-compulsive disorder
MDD: Major Depressive Disorder
GAD: Generalized Anxiety Disorder
HAM-D: Hamilton Rating Scale for Depression
BDS: Beirut Distress Scale
KMO: Kaiser-Meyer-Olkin
MANCOVA: Multivariate analysis of covariance
