# Supplementary material for: Association between impulsivity and orthorexia nervosa / healthy orthorexia: any mediating effect of depression, anxiety, and stress?
Source: BMC Psychiatry. 2021 Dec 3;21:604. doi: 10.1186/s12888-021-03594-4 (PMC8640965; doi:10.1186/s12888-021-03594-4)
Supplement: Supplementary file 1 — Additional file 1. [file 12888_2021_3594_MOESM1_ESM.docx]

| **Supplementary table 1. Mediation analysis.** | | | | | | | | | | | | | | | | |
| --- | --- | --- | --- | --- | --- | --- | --- | --- | --- | --- | --- | --- | --- | --- | --- | --- |
|  | | | | | | | | | | | | | | | | |
| **Model 1: Impulsivity, depression and orthorexia nervosa.** | | | | | | | | | | | | | | | | |
|  | Effect of impulsivity on depression | | | | | Effect of impulsivity and depression on orthorexia nervosa | | | | | Direct effect of impulsivity on orthorexia nervosa | | | | | Mediating effect of depression |
|  | Beta | 95% BCa CI | | t | p | Beta | 95% BCa CI | | t | p | Beta | 95% BCa CI | | t | p |  |
| Impulsivity | 0.12 | 0.01 | 0.23 | 2.21 | **0.027** | -0.06 | -0.12 | 0.03 | -1.39 | 0.166 | -0.08 | -0.17 | 0.01 | -1.71 | 0.088 | - |
| Depression |  |  |  |  |  | -0.13 | -0.20 | -0.05 | -3.23 | **0.001** |  |  |  |  |  |  |
|  | | | | | | | | | | | | | | | | |
| Urgency | -0.17 | -0.67 | 0.33 | -0.67 | 0.503 | 0.22 | -0.20 | 0.64 | 1.03 | 0.302 | 0.24 | -0.18 | 0.66 | 1.12 | 0.261 | - |
| Depression |  |  |  |  |  | -0.13 | -0.20 | -0.05 | -3.30 | **0.001** |  |  |  |  |  |  |
|  | | | | | | | | | | | | | | | | |
| Premeditation | 0.76 | 0.20 | 1.32 | 2.65 | **0.008** | -0.38 | -0.86 | 0.10 | -1.55 | 0.12 | -0.47 | -0.95 | 0.01 | -1.94 | 0.053 | - |
| Depression |  |  |  |  |  | -0.12 | -0.20 | -0.05 | -3.15 | **0.001** |  |  |  |  |  |  |
|  | | | | | | | | | | | | | | | | |
| Perseverance | 0.43 | -0.09 | 0.96 | 1.62 | 0.104 | -0.62 | -1.06 | -0.18 | -2.76 | **0.006** | -0.67 | -1.11 | -0.22 | -2.97 | **0.003** | 8.08% |
| Depression |  |  |  |  |  | -0.12 | -0.19 | -0.04 | -2.94 | **0.003** |  |  |  |  |  |  |
|  | | | | | | | | | | | | | | | | |
| Sensation seeking | 0.42 | -0.14 | 0.98 | 1.48 | 0.139 | -0.16 | -0.64 | 0.31 | -0.69 | 0.491 | -0.22 | -0.70 | 0.25 | -0.91 | 0.362 | - |
| Depression |  |  |  |  |  | -0.13 | -0.21 | -0.06 | -3.38 | **<0.001** |  |  |  |  |  |  |
|  | | | | | | | | | | | | | | | | |
| **Model 2: Impulsivity, anxiety and orthorexia nervosa.** | | | | | | | | | | | | | | | | |
|  | Effect of impulsivity on anxiety | | | | | Effect of impulsivity and anxiety on orthorexia nervosa | | | | | Direct effect of impulsivity on orthorexia nervosa | | | | | Mediating effect of anxiety |
|  | Beta | 95% BCa CI | | t | p | Beta | 95% BCa CI | | t | p | Beta | 95% BCa CI | | t | p |  |
| Impulsivity | -0.10 | -0.25 | 0.06 | -1.24 | 0.214 | -0.06 | -0.15 | 0.03 | -1.39 | 0.166 | -0.08 | -0.17 | 0.01 | -1.69 | 0.092 | - |
| Anxiety |  |  |  |  |  | 0.18 | 0.12 | 0.23 | 6.44 | **<0.001** |  |  |  |  |  |  |
|  | | | | | | | | | | | | | | | | |
| Urgency | 0.71 | 0.01 | 1.41 | 1.99 | **0.046** | 0.22 | -0.20 | 0.64 | 1.03 | 0.302 | 0.34 | -0.09 | 0.78 | 1.56 | 0.118 | - |
| Anxiety |  |  |  |  |  | 0.18 | 0.12 | 0.23 | 6.38 | **<0.001** |  |  |  |  |  |  |
|  | | | | | | | | | | | | | | | | |
| Premeditation | -0.51 | -1.31 | 0.29 | -1.25 | 0.211 | -0.38 | -0.86 | 0.10 | -1.55 | 0.121 | -0.47 | -0.97 | 0.03 | -1.85 | 0.064 | - |
| Anxiety |  |  |  |  |  | 0.18 | 0.12 | 0.23 | 6.41 | **<0.001** |  |  |  |  |  |  |
|  | | | | | | | | | | | | | | | | |
| Perseverance | -0.52 | -1.26 | 0.22 | -1.38 | 0.167 | -0.62 | -1.06 | -0.18 | -2.76 | **0.006** | -0.71 | -1.17 | -0.25 | -3.05 | **0.002** | 14.65% |
| Anxiety |  |  |  |  |  | 0.17 | 0.12 | 0.23 | 6.25 | **<0.001** |  |  |  |  |  |  |
|  | | | | | | | | | | | | | | | | |
| Sensation seeking | -0.86 | -1.64 | -0.07 | -2.15 | **0.031** | -0.16 | -0.64 | 0.31 | -0.69 | 0.491 | -0.32 | -0.81 | 0.17 | -1.28 | 0.201 | - |
| Anxiety |  |  |  |  |  | 0.18 | 0.12 | 0.23 | 6.38 | **<0.001** |  |  |  |  |  |  |
|  | | | | | | | | | | | | | | | | |
| **Model 3: Impulsivity, stress and orthorexia nervosa.** | | | | | | | | | | | | | | | | |
|  | Effect of impulsivity on stress | | | | | Effect of impulsivity and stress on orthorexia nervosa | | | | | Direct effect of impulsivity on orthorexia nervosa | | | | | Mediating effect of stress |
|  | Beta | 95% BCa CI | | t | p | Beta | 95% BCa CI | | t | p | Beta | 95% BCa CI | | t | p |  |
| Impulsivity | -0.08 | -0.15 | -0.01 | -2.15 | **0.032** | -0.06 | -0.15 | 0.03 | -1.39 | 0.166 | -0.06 | -0.15 | 0.03 | -1.38 | 0.167 | - |
| Stress |  |  |  |  |  | -0.01 | -0.12 | 0.11 | -0.13 | 0.895 |  |  |  |  |  |  |
|  | | | | | | | | | | | | | | | | |
| Urgency | 0.71 | 0.38 | 1.03 | 4.25 | **<0.001** | 0.22 | -0.20 | 0.64 | 1.03 | 0.302 | 0.21 | -0.20 | 0.62 | 1.01 | 0.311 | - |
| Stress |  |  |  |  |  | -0.1 | -0.13 | 0.10 | -0.20 | 0.840 |  |  |  |  |  |  |
|  | | | | | | | | | | | | | | | | |
| Premeditation | -0.87 | -1.24 | -0.50 | -4.62 | **<0.001** | -0.38 | -0.86 | 0.10 | -1.55 | 0.121 | -0.37 | -0.84 | 0.10 | -1.56 | 0.120 | - |
| Stress |  |  |  |  |  | -0.01 | -0.12 | 0.11 | -0.15 | 0.882 |  |  |  |  |  |  |
|  | | | | | | | | | | | | | | | | |
| Perseverance | -0.59 | -0.94 | -0.24 | -3.29 | **0.001** | -0.62 | -1.06 | -0.18 | -2.76 | **0.006** | -061 | -1.05 | -0.18 | -2.76 | **0.006** | - |
| Stress |  |  |  |  |  | -0.02 | -0.13 | 0.10 | -0.26 | 0.794 |  |  |  |  |  |  |
|  | | | | | | | | | | | | | | | | |
| Sensation seeking | -0.41 | -0.78 | -0.03 | -2.11 | **0.034** | -0.16 | -0.64 | 0.31 | -0.69 | 0.491 | -0.17 | -0.64 | 0.30 | -0.70 | 0.483 | - |
| Stress |  |  |  |  |  | 0.01 | -0.11 | 0.12 | 0.09 | 0.926 |  |  |  |  |  |  |

**Supplementary table 2. Mediation analysis.**

| **Model 1: Impulsivity, depression and healthy orthorexia.** | | | | | | | | | | | | | | | | |
| --- | --- | --- | --- | --- | --- | --- | --- | --- | --- | --- | --- | --- | --- | --- | --- | --- |
|  | Effect of impulsivity on depression | | | | | Effect of impulsivity and depression on healthy orthorexia | | | | | Direct effect of impulsivity on healthy orthorexia | | | | | Mediating effect of depression |
|  | Beta | 95% BCa CI | | t | p | Beta | 95% BCa CI | | t | p | Beta | 95% BCa CI | | t | p |  |
| Urgency | 0.001 | -0.51 | 0.51 | 0.004 | 0.997 | -0.61 | -1.08 | -0.01 | -2.57 | **0.010** | -0.61 | -1.08 | -0.14 | -2.57 | **0.010** | - |
| Depression |  |  |  |  |  | -0.04 | -0.13 | 0.04 | -1.03 | 0.301 |  |  |  |  |  |  |
|  | | | | | | | | | | | | | | | | |
| Premeditation | 0.63 | 0.05 | 1.20 | 2.15 | **0.032** | 0.30 | -0.23 | 0.84 | 1.12 | 0.263 | 0.27 | -0.26 | 0.80 | 1.01 | 0.313 | - |
| Depression |  |  |  |  |  | -0.05 | -0.14 | 0.04 | -1.14 | 0.255 |  |  |  |  |  |  |
|  | | | | | | | | | | | | | | | | |
| Perseverance | 0.38 | -0.11 | 0.87 | 1.53 | 0.126 | 0.38 | -0.11 | 0.87 | 1.53 | 0.126 | 0.37 | -0.12 | 0.86 | 1.48 | 0.138 | - |
| Depression |  |  |  |  |  | -0.03 | -0.12 | 0.05 | -0.76 | 0.449 |  |  |  |  |  |  |
|  | | | | | | | | | | | | | | | | |
| Sensation seeking | 0.61 | 0.05 | 1.18 | 2.13 | **0.033** | -0.10 | -0.62 | 0.42 | -0.37 | 0.71 | -0.12 | -0.64 | 0.40 | -0.45 | 0.648 | - |
| Depression |  |  |  |  |  | -0.04 | -0.12 | 0.05 | -0.85 | 0.396 |  |  |  |  |  |  |
|  | | | | | | | | | | | | | | | | |
| **Model 2: Impulsivity, anxiety and healthy orthorexia.** | | | | | | | | | | | | | | | | |
|  | Effect of impulsivity on anxiety | | | | | Effect of impulsivity and anxiety on healthy orthorexia | | | | | Direct effect of impulsivity on healthy orthorexia | | | | | Mediating effect of anxiety |
|  | Beta | 95% BCa CI | | t | p | Beta | 95% BCa CI | | t | p | Beta | 95% BCa CI | | t | p |  |
| Urgency | 0.68 | -0.03 | 1.40 | 1.88 | 0.06 | -0.61 | -1.08 | -0.14 | -2.57 | **0.01** | -0.52 | -1.00 | -0.04 | -2.15 | **0.032** | 14.96% |
| Anxiety |  |  |  |  |  | 0.13 | 0.07 | 0.19 | 4.33 | **<0.001** |  |  |  |  |  |  |
| 0.28 0.08 0.05 | | | | | | | | | | | | | | | | |
| Premeditation | -0.54 | -1.35 | 0.27 | -1.31 | 0.190 | 0.30 | -0.23 | 0.84 | 1.12 | 0.263 | 0.23 | -0.31 | 0.78 | 0.85 | 0.395 | - |
| Anxiety |  |  |  |  |  | 0.13 | 0.07 | 0.19 | 4.12 | **<0.001** |  |  |  |  |  |  |
|  | | | | | | | | | | | | | | | | |
| Perseverance | -0.54 | -1.29 | 0.21 | -1.42 | 0.156 | 0.38 | -0.11 | 0.87 | 1.53 | 0.126 | 0.31 | -0.19 | 0.81 | 1.24 | 0.217 | - |
| Anxiety |  |  |  |  |  | 0.13 | 0.07 | 0.19 | 4.10 | **<0.001** |  |  |  |  |  |  |
|  | | | | | | | | | | | | | | | | |
| Sensation seeking | -0.95 | -1.74 | -0.16 | -2.37 | **0.018** | -0.10 | -0.62 | 0.42 | -0.37 | 0.712 | -0.21 | -0.74 | 0.32 | -0.79 | 0.430 | - |
| Anxiety |  |  |  |  |  | 0.12 | 0.06 | 0.18 | 3.83 | **<0.001** |  |  |  |  |  |  |
|  | | | | | | | | | | | | | | | | |
| **Model 3: Impulsivity, stress and healthy orthorexia.** | | | | | | | | | | | | | | | | |
|  | Effect of impulsivity on stress | | | | | Effect of impulsivity and stress on healthy orthorexia | | | | | Direct effect of impulsivity on healthy orthorexia | | | | | Mediating effect of stress |
|  | Beta | 95% BCa CI | | t | p | Beta | 95% BCa CI | | t | p | Beta | 95% BCa CI | | t | p |  |
| Urgency | 0.61 | 0.27 | 0.94 | 3.55 | **<0.001** | -0.61 | -1.08 | -0.14 | -2.57 | **0.01** | -0.70 | -1.17 | -0.24 | -2.98 | **0.003** | 15.12% |
| Stress |  |  |  |  |  | -0.15 | -0.28 | -0.02 | -2.34 | **0.019** |  |  |  |  |  |  |
|  | | | | | | | | | | | | | | | | |
| Premeditation | -0.79 | -1.17 | -0.42 | -4.14 | **<0.001** | 0.30 | -0.23 | 0.84 | 1.12 | 0.263 | 0.44 | -0.09 | 0.96 | 1.63 | 0.104 | - |
| Stress |  |  |  |  |  | -0.17 | -0.30 | -0.04 | -2.52 | **0.012** |  |  |  |  |  |  |
|  | | | | | | | | | | | | | | | | |
| Perseverance | -0.48 | -0.84 | -0.12 | -2.65 | **0.008** | 0.38 | -0.11 | 0.87 | 1.53 | 0.126 | 0.46 | -0.03 | 0.95 | 186 | 0.063 | - |
| Stress |  |  |  |  |  | -0.17 | -0.30 | -0.04 | -2.60 | **0.009** |  |  |  |  |  |  |
|  | | | | | | | | | | | | | | | | |
| Sensation seeking | -0.46 | -0.83 | -0.08 | -2.36 | **0.019** | -0.10 | -0.62 | 0.42 | -0.37 | 0.712 | -0.02 | -0.54 | 0.50 | -0.07 | 0.944 | - |
| Stress |  |  |  |  |  | -0.17 | -0.30 | -0.05 | -2.69 | **0.007** |  |  |  |  |  |  |
